# More Dental Chemistry

**Published:** 1858-10

**Authors:** 


*More Dental Chemistry.*—At the recent meeting of the Dental Convention, the interesting question of the origin of caries again came up, and again the unfounded theories about nitric acid in the mouth played a conspicuous part in the attempt at explanation. It was said that nitric acid produced upon teeth out of the body, appearances resembling white decay, that nitric acid may be found in the mouth, and therefore we may infer that nitric acid is the sole agent in the production of the white decay.

Now all this is purely hypothetical, and rests upon no basis of connected observation. For, in order to prove this particular reagent in the cause of the decay, it must first be proved that it is actually present; not merely that it may occur. The chemist who experiments upon digestion out of the body and finds that certain acids will accomplish the solution of ordinary food, has no right to reason from this observation to the action of the stomach itself, and claim a discovery of the true physiological solvent. He must absolutely demonstrate the presence, in the gastric juice, of the free acid which he claims to be the agent. Until he does that, he is merely wandering among probabilities, which any man of common ingenuity can do till the end of time, without, in the smallest degree, furthering the progress of science.

There are, indeed, very good reasons for doubting the presence of free nitric acid in the mouth. The hypothesis of the conversion of ammonia into nitric acid will not answer at all. The conditions under which this change takes place do not exist in the mouth, and the surrounding deoxidating influences render it almost impossible that it could occur. It is no easy matter, as every chemist who has tried the experiment very well knows, to accomplish this result when the ammonia is pure and all the concurrent circumstances are arranged especially to produce it.

Nitric acid in a free state is formed in nature, according to Bischoff, only when an electric spark passes through oxygen and nitrogen. In combination it is repeatedly formed when putrefaction of organic matters takes place in contact with alkalies, but the result is an alkaline salt and not a free acid. It is notorious that nearly all the nitrogen of organic compounds passes off, during putrefaction, in the form of carbonate of ammonia, carbonic acid being the final result of the oxidation of the carbon with which these substances abound. Schlieper & Guckelberger, who examined with great care the products of putrefaction of animal matters, found that with the exception of hydrocyanic acid, all the acids formed during this decomposition were devoid of nitrogen. Other observers deny even the presence of hydrocyanic acid; so that if they are correct, not only is nitric acid not formed in this process, but not even any acid which has nitrogen for a component part.

These are the objections to the hypothesis of the formation of free nitric acid in the mouth, to dissolve out the phosphate of lime of the decaying tooth. If any one still believes in its existence, let him prove it by undoubted chemical tests. One well established fact is worth a thousand speculations. The latter may astonish admiring novices, but are of no value whatever as contributions to science.

It is to be hoped we shall soon get rid of such idle conjectures and get some definite basis of facts.

